# Inhibition of CXCR4 and CXCR7 Is Protective in Acute Peritoneal Inflammation

**DOI:** 10.3389/fimmu.2020.00407

**Published:** 2020-03-10

**Authors:** Kristian-Christos Ngamsri, Christoph Jans, Rizki A. Putri, Katharina Schindler, Jutta Gamper-Tsigaras, Claudia Eggstein, David Köhler, Franziska M. Konrad

**Affiliations:** Department of Anesthesiology and Intensive Care Medicine, University Hospital of Tübingen, Tübingen, Germany

**Keywords:** PMN, neutrophil, SDF-1, stromal cell-derived factor, tight junction proteins, adenosine receptor A_**2B**_

## Abstract

Our previous studies revealed a pivotal role of the chemokine stromal cell-derived factor (SDF)-1 and its receptors CXCR4 and CXCR7 on migratory behavior of polymorphonuclear granulocytes (PMNs) in pulmonary inflammation. Thereby, the SDF-1-CXCR4/CXCR7-axis was linked with adenosine signaling. However, the role of the SDF-1 receptors CXCR4 and CXCR7 in acute inflammatory peritonitis and peritonitis-related sepsis still remained unknown. The presented study provides new insight on the mechanism of a selective inhibition of CXCR4 (AMD3100) and CXCR7 (CCX771) in two models of peritonitis and peritonitis-related sepsis by injection of zymosan and fecal solution. We observed an increased expression of SDF-1, CXCR4, and CXCR7 in peritoneal tissue and various organs during acute inflammatory peritonitis. Selective inhibition of CXCR4 and CXCR7 reduced PMN accumulation in the peritoneal fluid and infiltration of neutrophils in lung and liver tissue in both models. Both inhibitors had no anti-inflammatory effects in A_2B_ knockout animals (A_2B_–/–). AMD3100 and CCX771 treatment reduced capillary leakage and increased formation of tight junctions as a marker for microvascular permeability in wild type animals. In contrast, both inhibitors failed to improve capillary leakage in A_2B_–/– animals, highlighting the impact of the A_2B_-receptor in SDF-1 mediated signaling. After inflammation, the CXCR4 and CXCR7 antagonist induced an enhanced expression of the protective A_2B_ adenosine receptor and an increased activation of cAMP (cyclic adenosine mono phosphate) response element-binding protein (CREB), as downstream signaling pathway of A_2B_. The CXCR4- and CXCR7-inhibitor reduced the release of cytokines in wild type animals via decreased intracellular phosphorylation of ERK and NFκB p65. *In vitro*, CXCR4 and CXCR7 antagonism diminished the chemokine release of human cells and increased cellular integrity by enhancing the expression of tight junctions. These protective effects were linked with functional A_2B_-receptor signaling, confirming our *in vivo* data. In conclusion, our study revealed new protective aspects of the pharmacological modulation of the SDF-1-CXCR4/CXCR7-axis during acute peritoneal inflammation in terms of the two hallmarks PMN migration and barrier integrity. Both anti-inflammatory effects were linked with functional adenosine A_2B_-receptor signaling.

## Introduction

Peritonitis and peritonitis-related sepsis are still associated with a high mortality for up to 40–60% (1). In the United States, sepsis is more common than myocardial infarction or colon cancer (2, 3). Despite decades of research, the underlying mechanisms are still not understood and therefore, there is still no functional treatment of sepsis possible (4–6). Sepsis is caused by an overshooting answer of the immune system on the infection, resulting in injuring its own organs. This acute pro-inflammatory response of the body is mainly driven by polymorphonuclear neutrophils (PMNs) as the first cells of the immune system to be recruited to the side of inflammation (7, 8). Accordingly, PMNs are considered as a prognostic marker for mortality in terms of sepsis (9) as they migrate from the circulatory system into the inflamed tissue.

Beside PMN migration, the second hallmark of sepsis is capillary leakage (10). Tight junction proteins (TJP) are intracellular adhesion complexes controlling paracellular permeability and are therefore involved in maintaining tissue homeostasis (11). More precisely, TJPs are located apically in polarized cells and regulate the passage of water, ions and molecules (12, 13) and are also involved in cellular signaling (14). Inflammation and hypoxia alter the integrity of the tissue and paracellular permeability (15–18), leading to the clinically observed tissue edema (19, 20).

In case of inflammation, the chemokine stromal cell-derived factor (SDF)-1 in the bone marrow decreases and PMNs are released into the vasculature to migrate to inflamed areas (21). SDF-1 has two receptors—CXCR4 and CXCR7—both widely expressed on hematopoietic and non-hematopoietic cells (22–24). Both SDF-1-receptors drive endothelial and epithelial transmigration of leukocytes during acute inflammation (23, 25, 26). Pharmacological inhibition of CXCR4 protects lung tissue and keeps tissue homeostasis during acute and chronic pulmonary inflammation by reducing infiltration of PMNs, respectively CXCR4-positive cells (25, 27). CXCR4 blockade improved stroke-related damage and reduced the blood-brain barrier disruption by reducing the release of inflammatory cytokines in the ischemic region (28). Also, CXCR7 regulated acute inflammatory and allergic-related edema formation by stabilizing the pulmonary epithelial barrier (29, 30).

Recent literature linked SDF-1 related signaling to a functional adenosine A_2B_-receptor (25, 31). The nucleoside adenosine exerts its functions through four different adenosine receptors. The cell surface G protein-coupled adenosine receptors A_1_, A_2A_, A_2B_, and A_3_ play a central role in various inflammatory diseases (15, 32–34). Activation of the A_2B_-receptor plays a protective role in terms of tissue homeostasis and maintaining cellular barrier function during inflammation (15, 32, 35).

In contrast, patients on the intensive care unit (ICU) reveal altered expression of adenosine receptors and compromised ligand affinity (36, 37). Accordingly, if therapy of sepsis is linked to functional adenosine receptor signaling, the expression level and the functionality of the receptors should be evaluated to adapt and elaborate an individualized therapy.

Current literature demands the identification of subgroups of patients for a customized therapy (38, 39). In the presented study, we investigated the specific role of the SDF-1 receptors CXCR4 and CXCR7 during acute inflammatory peritonitis and peritonitis-related sepsis concerning the two hallmarks of acute inflammation, migration of PMNs and barrier permeability. Additional, we hypothesized that the protection through CXCR4 and CXCR7 antagonism depends on functional A_2B_-receptors. To enlarge the impact of our study, we determined these aspects in a zymosan- and additionally in fecal-induced peritonitis.

## Materials and Methods

### Animals

Mice were housed under pathogen-free conditions and on standard light-dark cycle. Mice were male and 8–12 weeks old (wild type: C57BL/6N; Charles River; Germany; A_2B_ knockout mice: A_2B_–/–; kindly gift from Dr. Katya Ravid; Boston University; School of Medicine; Department of Biochemistry; USA). All animal protocols were approved by the Animal Care and Use Committee of the University of Tübingen.

### Reagents

CCX771, the specific CXCR7 antagonist (10 mg/kg body weight [bw]; ChemoCentryx; USA), was injected subcutaneously and the specific CXCR4 antagonist AMD3100 (10 mg/kg bw; Sigma Aldrich; Germany) was administrated intraperitoneally (i.p.) 1 h before zymosan application (zymosan-A of Saccharomyces cerevisiae; 50 mg/kg bw; i.p. injection; Sigma-Aldrich; Germany).

### Zymosan-Induced Peritonitis and Sepsis

Peritoneal inflammation was induced by zymosan application i.p. (1 mg per mouse; concentration: 1 mg/ml). Four hours after zymosan administration, 5 ml of PBS- were injected into the peritoneal cavity and 3 ml peritoneal fluid lavage were retrieved. After thoracotomy, blood samples were collected by right ventricle punctuation and the vascular system was flushed by 3 ml PBS- for blood-free organs. Peritoneal lavage and tissue (lung and liver) samples were removed for flow cytometry analysis and partly saved for subsequent experiments at −80°C.

### Fecal-Induced Peritonitis and Sepsis

To prepare the fecal solution, we collected fecal dry pellets randomly from C57BL/6N male mice cages with same age and diet. Fecal material was pooled, diluted with normal saline to a concentration of 80 mg/ml, aliquoted and the same fecal stock solution used for this whole project. The fecal solution was injected intraperitoneally. After 4 h, peritoneal lavage, blood and organs were collected as described above.

### RT-PCR

Total RNA was isolated from murine peritoneum, lungs and liver by using pegGOLD TriFast (Peqlab, Germany), and cDNA synthesis was performed by using a Bio-Rad iScript kit (Bio-Rad, Germany) according to the manufacturer's directions. We evaluated the gene expression of murine SDF-1, CXCR4, CXCR7, A_1_ adenosine receptor, A_2A_ adenosine receptor, A_3_ adenosine receptor, A_2B_ adenosine receptor, CD73, TNFα, and IL6 by using RT-PCR and the following primers: SDF-1 (5′-GAG AGC CAC ATC GCC AGA G-3′ and 5′-TTT CGG GTC AAT GCA CAC TTG-3′), CXCR4 (5′-AGC ATG ACG GAC AAG TAC C-3′ and 5′-GAT GAT ATG GAC AGC CTT ACA C-3′), CXCR7 (5′-GGA GCC TGC AGC GCT CAC CG-3′ and 5′-CTT AGC CTG GAT ATT CAC CC-3′), A_1_ (5′-ATT GTC ACT CAG CTC CCG C-3′ and 5′-TCA CCA GTA CAT TTC CGG GC-3′), A_2A_ (5′-TCA ACA GCA ACC TGC AGA AC-3′ and 5′-GGC TGA AGA TGG AAC TCT GC-3′), A_2B_ (5′-GCG TCC CGC TCA GGT ATA AA-3′ and 5′-CAG TGG AGG AAG GAC ACA CC-3′), A_3_ (5′-GGG TTC CTG TAC TTC CTC TTG G-3′ and 5′-TCA ACC TCA GCC GCT TAT CC-3′), CD73 (5′-GTT CTC TCT GTT GGC GGT G-3′ and 5′-GGA TGC CAC CTC CGT TTA C-3′), TNFα (5′-GGA GCC TGC AGC GCT CAC CG-3′ and 5′-CTT AGC CTG GAT ATT CAC CC-3′), and IL6 (5′-GGA GCC TGC AGC GCT CAC CG-3′ and 5′-CTT AGC CTG GAT ATT CAC CC-3′). Gene levels of barrier integrity related proteins were evaluated by utilizing subsequent primers for murine occludin (OCLDN), tight junctions proteins 1–3 (TJP 1, 2, and 3), e-cadherin 1 (CDH1), and claudin (CLDN) 1, 3, and 5: OCLDN (5′- GTG GGA TAA GGA ACA CAT TT-3′ and 5′-GAC ACA TTT TTA ACC CAC TC-3′), TJP1 (5′-CCT TGG CCT AGC ATA CAC A-3′ and 5′-GAA ATC GTG CTG ATG TGC C- 3′), TJP2 (5′-CAG CAA GCA GAC CCT CAT C-3′ and 5′- TCC AGC TCA TTC CCG ATC C- 3′), TJP3 (5′-CGA CTA TGA GGA CAC CGA C-3′ and 5′-TGT CCC ATG ACC CAT CAG C- 3′), CDH1 (5′-CAG CTC CTT CCC TGA GTG-3′ and 5′-GCA CCC ACA CCA AGA TAC-3′), CLDN1 (5′- CCA CCA TTG GCA TGA AGT GC-3′ and 5′-AGA GGT TGT TTT CCG GGG AC-3′), CLDN3 (5′-CCT ACG ACC GCA AGG ACT AC-3′ and 5′- CTG GTA GTG GTG ACG GTA CG-3′), CLDN5 (5′- CCA CCA TTG GCA TGA AGT GC-3′ and 5′-AGA GGT TGT TTT CCG GGG AC-3′).

To reveal the gene expression of the human adenosine A_2B_-receptor, IL6, and IL8, we used the following primers and performed RT-PCR: A_2B_ (5′-ATC TCC AGG TAT CTT CTC-3′ and 5′-GTT GGC ATA ATC CAC ACA G-3′), IL6 (5′-CCA CCA TCT ACT CCA TCA TCT TC-3′ and 5′-ACT TGT CCG TCA TGC TTC TC-3′), and IL8 (5′-AGC ACA GCC AGG AAG GCG AG-3′ and 5′-TCA TAG CCT GTG TTG GC-3′).

18S was used as house keeping gene (5′-GTA ACC CGT TGA ACC CCA TT-3′ and 5′-CCA TCC AAT CGG TAG TAG CG-3′).

### Microvascular Permeability

Protein extravasation into the peritoneal lavage as a marker of capillary leakage was determined 4 h after zymosan, respectively 4 h after fecal-injection by using a BCA protein assay kit according to the standard protocol (Pierce; Thermo Fisher Scientific; Germany). Endothelial leakage was assessed by fluorescein isothiocyanate conjugated albumin (FITC-albumin; A9771; Sigma-Aldrich) extravasation in separate experiments. FITC-albumin (80 mg/kg BW) was injected into the tail vein 30 min before removal of peritoneal lavage. FITC-albumin concentration was measured in the lavage.

### Cytokine Concentrations

The release of TNFα, IL6, CXCL1 (keratinocyte-derived chemokine), CXCL2/3 (macrophage inflammatory protein-2), and SDF-1α was determined in the peritoneal lavage of mice, 4 h after zymosan- and fecal-injection by ELISA kits (DY406; DY453; DY452; DY410, and DY460; R&D Systems; USA). Zymosan-induced release of IL6 and IL8 by human H441 cells was also detected by ELISA kits (DY206, respectively DY208; R&D Systems; USA).

### *In vivo* PMN Extravasation

As previously described, lungs and liver samples were homogenized and prepared for flow cytometer staining procedure (25, 40). Peritoneal lavage (PL), lungs and liver samples were stained with a fluorescent antibody-mix, consisting of CD45 (clone 30-F11; 103132; BioLegend; USA) and Ly6G (clone 1A8; 127618; BioLegend; USA) to detect PMNs. The detailed description of the gating process is described in Supplemental Figure 1A. Samples were measured with a FACSCanto II flow cytometer (BD Biosciences; USA). The cytometer was calibrated routinely using the cytometer setup and tracking beads (BD Biosciences; USA) recommended by the manufacturer. BD FACSDiva software (Version 6; BD Biosciences; USA) was employed to control the flow cytometer settings, including the calibration procedures, and to acquire data. Detailed data analysis was performed using FlowJo software (version 7.8.2; Ashland; USA).

### Western Blot Analysis

Mice were treated as described above and peritoneal tissue from wild type and A_2B_–/– animals prepared for western blot analysis. Equivalent protein levels were determined by a protein assay kit (Pierce; Thermo Fisher Scientific; Germany) and loaded on SDS gels. After blotting on polyvinyldene difluoride membranes, the rabbit polyclonal anti–phospho NF-κB p65 (Ser536)(#3033; Cell Signaling Technology; Germany), the rabbit polyclonal anti–phospho ERK1/2 (Thr202/Tyr204) (#4370; Cell Signaling Technology; Germany) and the rabbit monoclonal anti-phospho CREB (Ser133) (#9198; Cell Signaling Technology; Germany) were used. For analyzing the impact of CXCR4 and CXCR7 on the formation of tight junctions 4 h after zymosan and autologous fecal administration, we used rabbit polyclonal anti–tight junction protein (TJP)-1 (1 mg/ml; Thermo Fisher Scientific; Germany) and mouse monoclonal anti-occludin (0.5 mg/ml; Thermo Fisher Scientific; Germany). The rabbit monoclonal anti-GAPDH served as housekeeping protein (G9545; Sigma-Aldrich; Germany).

### Tissue Culture

In absence of a human peritoneal epithelial cell line, a human pulmonary epithelial cell line (H441; NCI-H441; ATCC® HTB-174™) and a human intestinal epithelial cell line (CaCo2; ATCC® HTB-37™) was used. H441 and CaCo2 cells were maintained in RPMI containing 10%FCS and 40 μg/ml gentamicin in a humidified atmosphere of 5% CO_2_ at 37°C. H441 and CaCo2 were grown confluent and stimulated with NaCl or zymosan 100 μg/ml for 4 h. Additional groups were treated with CCX771 (1 μM) or AMD3100 (1 μM) 1 h before zymosan administration. Supernatants were secured for protein analysis. Cells were removed and total RNA was isolated following the manufacturer's directions (pegGOLD TriFast; Peqlab; Germany and Bio-Rad iScript kit; Bio-Rad; Germany).

In additional experiments, we used siRNA to knock down the human adenosine receptor A_2B_ (sc-29642; Santa Cruz Biotechnology; USA) in H441 and CaCo2 cells. After the cell monolayer achieved 50% of confluence, medium was exchanged and cell layer transfected with jetPRIME® reagent (114-07; Polyplus transfection; France) and adenosine A_2B_ human siRNA added according to the manufacturer's instructions. 24 h after transfection, cells were harvested and total RNA was isolated for gene expression analysis. The success of siRNA transfection was evaluated by detection of gene levels of the adenosine receptor A_2B_ (Supplemental Figure 1B). Non-targeting siRNA (sc-37007; Santa Cruz Biotechnology; USA) was used as control.

### Immunofluorescence Staining

Paraffin-embedded lung sections were fixed for 10 min in acetone and methanol. After washing and fixation, lung sections were permeabilized with 1% Triton X-100 and blocked with 5% BSA in PBS for 1 h. Sections were stained with rabbit polyclonal anti-A_2B_ adenosine receptor (sc-28996; Santa Cruz Biotechnology; USA), goat polyclonal anti-CXCR7 (sc-107515; Santa Cruz Biotechnology; USA), rabbit polyclonal anti-CXCR4 (sc-9046; Santa Cruz Biotechnology; USA), rabbit monoclonal anti-phospho CREB (Ser133) (#9198; Cell Signaling Technology) and goat polyclonal anti-cytokeratin 12 (sc-17101; Santa Cruz Biotechnology; USA). For visualization, the following secondary antibodies were employed: polyclonal donkey anti-goat IgG Alexa Fluor 488 (A11055; Thermo Fisher Scientific; Germany), polyclonal goat anti-rabbit IgG Alexa Fluor488 (A11008; Thermo Fisher Scientific; Germany), and polyclonal rabbit anti-goat IgG Alexa Fluor 546 (A21085; Thermo Fisher Scientific; Germany). For nuclei counterstaining, we used Roti-Mount FluorCare DAPI (HP20.1; Carl Roth; Germany). IgG controls are displayed in Supplemental Figure 1C.

For *in vitro* immunofluorescence experiments, H441 and CaCo2 cells were grown on chamber slides (Sarstedt Neumbrecht; Germany). After stimulation for 4 h with 100 ng/ml zymosan with CXCR4- (AMD3100; 1 μM) or CXCR7-treatment (CCX771; 1 μM), cells were fixed with 4% paraformaldehyde. After permeabilization with 1% Triton X-100, cells were blocked for 1 h with 5% BSA in PBS. Cells were stained by using rabbit polyclonal anti–tight junction protein (TJP)-1 (1 mg/ml; Thermo Fisher Scientific; Germany) and mono l anti-occludin (0.5 mg/ml; Thermo Fisher Scientific; Germany) followed by the secondary antibodies as described above. Rhodamin phalloidin was used to tackle β-actin (R415; Thermo Fisher Scientific; Germany). Images were analyzed by using ZEN software (Black edition 2011; Zeiss; Germany) and mean fluorescence intensities were measured by ImageJ (Version 1.49v; National Institute of Health; USA).

### Immunohistochemical PMN Detection

PMN accumulation in peritoneal tissue, lung and liver sections was visualized via immunohistochemistry by using a Vectastain ABC kit (PK-4000; Vector Laboratories; Germany). Sections were blocked with Avidin solution (Vector Laboratories; Germany) for 1 h to avoid unspecific binding sites. PMNs were stained with rabbit anti-mouse Ly-6G (clone 1A8; Abcam; UK). Rabbit IgG was used as control (31235; Invitrogen; USA). Sections were incubated with biotinylated anti-rabbit IgG (BA-4000; Vector Laboratories; USA) for 1 h, followed by Vectastain ABC reagent (PK-4000; Vector Laboratories; USA) for 30 min and then incubated with DAB substrate. Nuclear fast red (H-3403; Linaris; Germany) was used for tissue counterstaining. Tissue slides were processed with a Leitz DM IRB microscope (Leica) and analyzed with AxioVision v4.8.2 (Carl Zeiss MicroImaging; Germany). Neutrophil counts were examined by enumerating the positive and therefore brown stained cells in a masked fashion. PMN numbers were scored from four random sections of four different tissue samples in each group by two independent observers (41).

### String Analysis

STRING is a biological database and free web resource to identify known and predicted protein-protein networks. The STRING database includes the information of numerous experimental data, various computational predictions, and public text data. Furthermore, the STRING analysis identifies molecular partnerships and functional interactions from targets of interest by consolidating knowledge and providing context in biological systems (42). To generate reliable results, we set a medium confidence (>0.400) over our analysis.

### Software and Statistical Analysis

Statistical analysis was performed by using Graph Pad Prism version 8.1 for Windows (Graph Pad Software; San Diego; USA). For comparison between two groups statistical analysis was done by an unpaired Student's *t*-test. Differences between the groups were evaluated by one-way ANOVA followed by Bonferroni's *post-hoc* test. Data are presented as mean ± SEM unless indicated otherwise.

## Results

### Expression of CXCR4 and CXCR7 in Acute Inflammation *in vivo*

**Figure 1:**
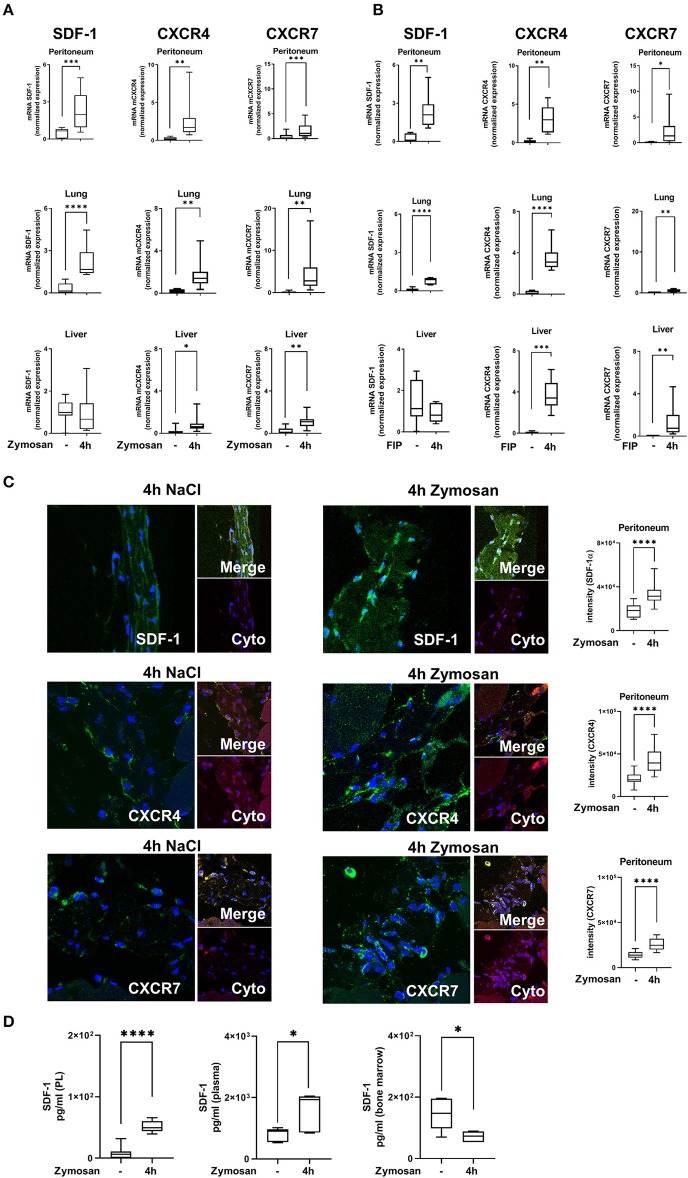
Expression of stromal cell-derived factor-1 (SDF-1) and its receptors CXCR4 and CXCR7 in acute inflammation. **(A)** SDF-1, CXCR4, and CXCR7 mRNA levels were evaluated in peritoneal, lung and liver tissue 4 h after zymosan (*n* = 8–12) or **(B)** fecal administration (*n* = 6–12). **(C)** Immunofluorescence detection of the expression of SDF-1, CXCR4, and CXCR7 (all green) in peritoneal tissue (Cyto; cytokeratin; red) 4 h after zymosan (63x original magnification). Images are representatives of *n* = 3 experiments. Intensity was measured by ImageJ. **(D)** The release of SDF-1α in the peritoneal cavity, in the plasma, and in the bone marrow of wild type mice was detected by using ELISA (*n* = 6–12). Student's *t*-test was used for statistical analysis. Data are presented as box and whisker graph with error bars indicating the range from minimum to maximum value; *p < 0.05; **p < 0.01; ***p < 0.001; ****p < 0.0001.

We evaluated gene expression of the chemokine SDF-1 and its receptors CXCR4 and CXCR7 in various organs and peritoneal tissue during zymosan- and fecal-induced peritonitis. We detected a significant mRNA increase of SDF-1 in the peritoneum and lung 4 h after zymosan- (Figure 1A) or fecal-administration (Figure 1B). Inflammation did not affect gene expression of SDF-1 in liver tissue. Gene expression of the two receptors CXCR4 and CXCR7 rose significantly after the onset of inflammation in the peritoneum, lung and liver tissue in both models. To verify these findings on protein level, we evaluated the surface expression of SDF-1, CXCR4, and CXCR7 in peritoneal tissue by immunofluorescence. According to the results of gene expression, protein levels of SDF-1 and both receptors increased after zymosan (Figure 1C). The release of SDF-1 in the peritoneal lavage, plasma and bone marrow was assessed by ELISA (Figure 1D). A significant rise of SDF-1 was observed in the plasma, respectively in the peritoneal lavage of WT animals. SDF-1 levels in the bone marrow were reduced, allowing the zymosan-induced mobilization of PMNs into the circulation.

### CXCR4- and CXCR7-Antagonism Controls PMN Migration in Acute Inflammation

**Figure 2:**
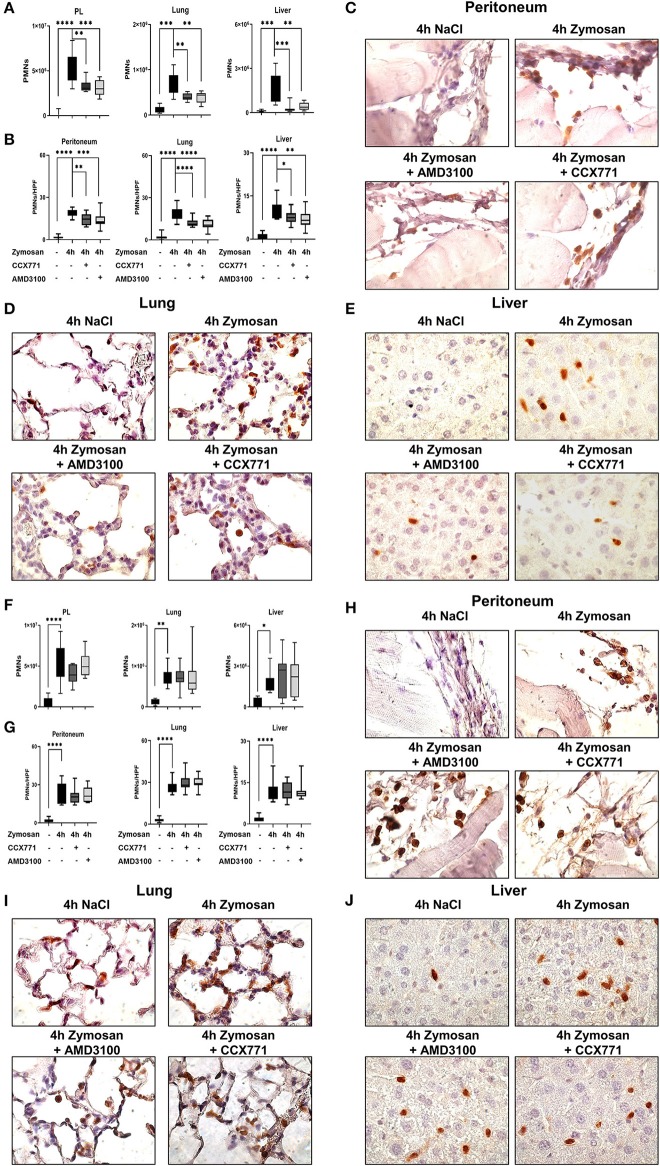
CXCR4 and CXCR7 treatment dampens neutrophil migration into inflamed tissue through A_2B_ purinergic signaling. **(A)** We evaluated the influx of polymorphonuclear leukocytes (PMNs) into the peritoneal lavage, lung and liver tissue in wild type (*n* = 8–12) and A_2B_–/– animals **(F)** (*n* = 6–12) with or without zymosan administration by flow cytometry. Animals were treated with AMD3100 (CXCR4-antagonist) or CCX771 (CXCR7-antagonist). PMN infiltration was also shown by immunohistochemistry, where PMNs were marked brown and counted per high power field **(B,G)** (*n* = 16). Representative histological examination of the **(C,H)** peritoneum, **(D,I)** lung and **(E,J)** liver tissue in wild type and A_2B_–/– animals are shown. Images are representatives of *n* = 3 experiments. For statistical analysis, one-way ANOVA was used with Bonferroni *post-hoc* test. Data are presented as box and whisker graph with error bars indicating the range from minimum to maximum value; *p < 0.05; **p < 0.01; ***p < 0.001 and ****p < 0.0001.

Zymosan-induced PMN migration into peritoneum, lung and liver tissue was evaluated by using a flow cytometry-based method. In wild type mice, zymosan increased the PMN influx into the peritoneal lavage, lung and liver tissue (Figure 2A). The inhibition of both receptors, CXCR4 and CXCR7, significantly reduced the infiltration of PMNs into the peritoneal cavity, lung and liver. To visualize these findings and determine the PMN infiltration into the organs quantitatively, we stained PMNs immunohistochemical so that they appear brown and evaluated these tissue sections blinded (Figure 2B). Four hours after zymosan application, PMN infiltration increased in all tissues compared to untreated animals (Figures 2C–E). Specific CXCR4 and CXCR7 inhibition reduced PMN infiltration in all tissues, confirming our data from flow cytometry. These results highlight the impact of CXCR4- and CXCR7-inhibition on the migratory behavior of PMNs during acute inflammation.

### The Anti-inflammatory Effects of CXCR4- and CXCR7-Inhibition Are Linked to a Functional A_2B_-Receptor

Following our hypothesis that the protective effects of CXCR4- and CXCR7-antagonism in acute peritonitis are linked to A_2B_-receptor signaling, we performed experiments with A_2B_–/– animals. Zymosan induced a significant rise of PMNs in the peritoneal lavage, lung and liver tissue (Figure 2F). In these knockout animals, the inhibition of CXCR4 and CXCR7 did not show any protective effects concerning PMN migration, neither in all tissues nor in the peritoneal lavage. Blinded evaluation of immunohistochemical slides on PMN counts confirmed our flow cytometry data (Figure 2G). PMN infiltration is represented in histological sections of the peritoneal (Figure 2H), lung (Figure 2I) and liver tissue (Figure 2J).

### CXCR4- and CXCR7-Inhibition Initiates Adenosine Receptor A_2B_ Signaling During Acute Inflammation

**Figure 3:**
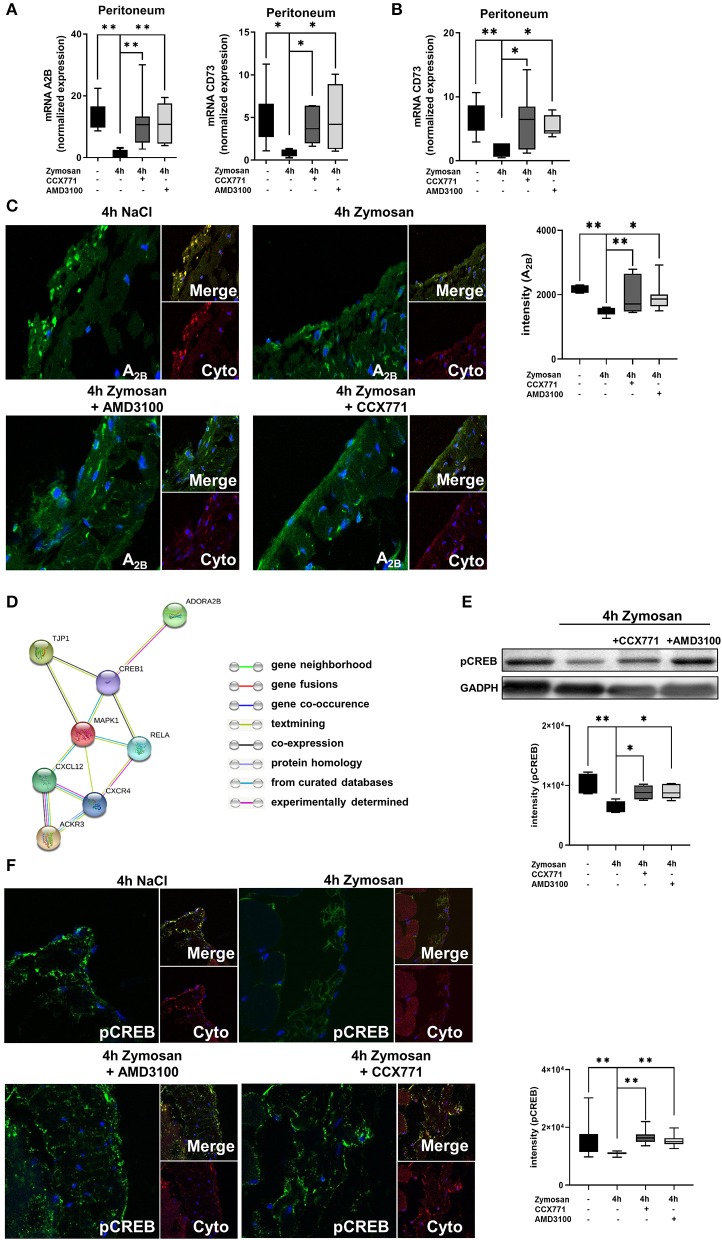
Link between the SDF-1-CXCR4-CXCR7-axis and the adenosine receptor A_2B_. **(A)** Expression levels of adenosine receptor A_2B_ and CD73 in peritoneal tissue 4 h after NaCl or zymosan with or without AMD3100 or CCX771 in wild type (WT) animals (*n* = 7–8). **(B)** Gene expression of CD73 in the peritoneum of A_2B_–/– mice at indicated conditions (*n* = 6–8). **(C)** Immunofluorescence staining of the A_2B_-expression (green) in peritoneal tissue (Cyto; cytokeratin red) (63x original magnification). Images are representatives of n=3 experiments. Adenosine receptor A_2B_ fluorescence intensity was assessed by using ImageJ. **(D)** Relationship between the SDF-1 (CXCL12)-CXCR4/CXCR7-axis, tight junction protein 1(TJP1), adenosine receptor A_2B_ (ADORA2B) and intracellular signaling proteins like NF-κB p65 (Rela), Mapk1 (ERK2), respectively cAMP response element-binding protein (CREB1) by String analysis. **(E)** Western blot analysis of the effects of CXCR4 and CXCR7 antagonism on the phosphorylation of intracellular CREB in peritoneal tissue of WT. Intensity of the blots were evaluated by ImageJ. **(F)** Detection of phosphoCREB (green) in peritoneal tissue of WT mice (Cyto; cytokeratin red) by using immunofluorescence staining (63x original magnification). Images are representatives of *n* = 3 experiments. Fluorescence intensity of phosphoCREB was assessed by using ImageJ. Data are presented as box and whisker graph with error bars indicating the range from minimum to maximum value; *n* = 6–12; *p < 0.05; **p < 0.01.

Our results demonstrated a link between the anti-inflammatory effects of the inhibition of both SDF-1 receptors and functional A_2B_-receptor signaling. Now, we investigated if the inhibition of CXCR4 and CXCR7 influences the expression of the A_2B_-receptor. After the application of zymosan, the expression of the A_2B_-receptor was significantly reduced in wild type animals and the inhibition of both receptors increased the expression again to baseline levels without inflammation (Figure 3A). To further verify this link and to exclude other influences from adenosine signaling, we also determined the expression of the ecto-5′-nucleotidase CD73. CD73 is critically involved in the generation of extracellular adenosine, which plays a pivotal role itself in acute inflammation (43). Comparable to the expression of A_2B_, CD73 was significantly reduced after the onset of inflammation and the inhibition of CXCR4 and CXCR7 increased the expression of the enzyme again to baseline levels. We also determined the expression in A_2B_–/– animals and observed the same result as in wild type animals, indicating that the A_2B_-receptor is the critical key in this setting (Figure 3B).

To confirm our data on protein level, we identified the surface expression of A_2B_ in the peritoneal tissue by immunofluorescence (Figure 3C). We detected a decrease of the expression of A_2B_ after zymosan, whereas CCX771 and AMD3100 elevated the surface expression of A_2B_ again.

The STRING analysis aims to collect, score and integrate all available knowledge of protein-protein interaction and to complement these with computational predictions of connections. By using STRING analysis, we searched for an association between the SDF-1-CXCR4/CXCR7-axis and the adenosine receptor A_2B_. STRING analysis showed a link between the SDF-1-CXCR4/CXCR7-axis and intracellular signaling proteins like RELA, MAPK1, and CREB1 with TJP1. Also, the adenosine receptor A_2B_ is linked with TJP1 through CREB1 (Figure 3D). The aim of this analysis was to provide a critical assessment of interactions from targets of interest. To get strong results, we set a medium confidence (>0.400) over our analysis.

To further investigate the connection between the adenosine receptor A_2B_, the phosphorylation of CREB and the SDF-1-CXCR4/CXCR7-axis in the setting of our study, we performed additional western blot experiments. We detected a significantly reduced activation of CREB in the peritoneal tissue after zymosan stimulation. Furthermore, the CXCR4 and CXCR7 inhibition augmented the activation of CREB (Figure 3E). Additionally, fluorescence studies confirmed our western blot results (Figure 3F). CREB is a downstream signaling pathway of A_2B_ and a cellular transcription factor. The activation of CREB initiates mainly anti-inflammatory effects, for example stabilization of tight junction proteins (44) and inhibiting NF-κB (45). The A_2B_ receptor is known to activate CREB (46). This increased phosphorylation of CREB through the adenosine receptor A_2B_ may explain the anti-inflammatory effects of the CXCR4, respectively CXCR7 inhibition on barrier integrity in the presented study.

To exclude any effects of the CXCR4 and CXCR7 inhibition on the adenosine receptors A_1_, A_2A_, and A_3_, we performed additional RT-PCR experiments. Four hours after zymosan, we observed a significant decrease of A_2A_ and A_3_ gene expression. A_1_ adenosine receptor expression did not alter during zymosan-induced peritonitis. The pharmacologic inhibition of CXCR4 and CXCR7 showed no effects on the expression of the adenosine receptors A_1_, A_2A_, and A_3_ (Supplemental Figure 2).

### Inhibition of CXCR4 and CXCR7 Stabilizes the Capillary Leakage

**Figure 4:**
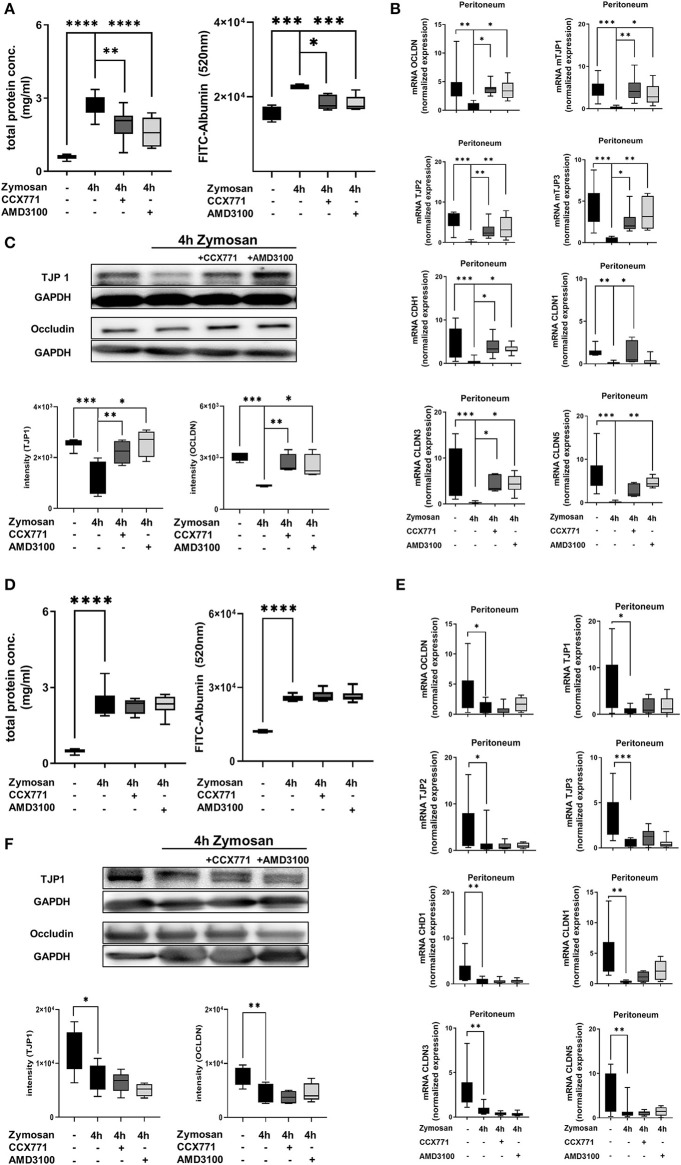
Influence of CXCR4 and CXCR7 on microvascular permeability and cellular integrity. **(A)** Protein accumulation (*n* = 6–12) and FITC-Albumin extravasation (*n* = 4–6) were evaluated in the peritoneal cavity 4 h after NaCl or zymosan injection in wild type and **(D)** A_2B_–/– animals. Effects of AMD3100 or CCX771 on capillary leakage were determined. **(B)** Gene levels of integrity-related tight junction proteins like occludin (OCLDN), tight junction protein 1, 2, and 3 (TJP1; TJP2; TJP3), e-cadherin 1 (CDH1), claudin 1, 3, and 5 (CLDN1; CLDN3; CLDN5) were measured in peritoneal tissue of wild type (*n* = 8–12) and **(E)** A_2B_–/– animals (*n* = 6–12). **(C)** Representative western blots of TJP1 and occludin protein of wild type and **(F)** A_2B_–/– peritoneal tissue are shown (representatives blots of *n* = 3 experiments). Intensity of the blots was evaluated by ImageJ. For statistical analysis, one-way ANOVA was used with Bonferroni *post-hoc* test. Data are presented as box and whisker graph with error bars indicating the range from minimum to maximum value; *p < 0.05; **p < 0.01; ***p < 0.001; ****p < 0.0001.

To evaluate the peritonitis-induced barrier destruction, we determined protein- and FITC-albumin extravasation into the peritoneal cavity. Zymosan-induced a significant increase of protein extravasation, respectively albumin, whereas the treatment of CCX771 and AMD3100 significantly reduced protein accumulation and therefore stabilized microvascular permeability (Figure 4A). For the endothelial and epithelial integrity, tight junction proteins are essential to control paracellular diffusion of water, ions and cells. Accordingly, we measured the expression of relevant tight junction proteins, like occludin (OCLDN), tight junction protein 1-3 (TJP1-3), e-cadherin (CDH1) and claudin1, 3, and 5 (CLDN1; CLDN3; CLDN5). We observed a significant depression of all analyzed tight junction proteins as inflammatory response in the peritoneum (Figure 4B). After the inhibition of CXCR4 and 7, we detected a significant elevation almost to baseline levels without inflammation of OCDLN, TJP1-3, CDH1, and CLDN3. The inhibition of CXCR7 resulted in a significant augmentation of CLDN1, whereas the inhibition of CXCR4 increased CLDN5. Western blot analysis from peritoneal tissue confirmed our above described findings for TJP1 and occludin (Figure 4C). Peritonitis reduced both tight junction proteins and the inhibition of CXCR4 and CXCR7 increased them again.

In A_2B_–/– animals, zymosan significantly increased protein extravasation and FITC-albumin accumulation into the peritoneal cavity (Figure 4D). CXCR4 and CXCR7 inhibition were unable to prevent capillary leakage and FITC-albumin extravasation in these knockout animals, highlighting again the pivotal role of a functional adenosine A_2B_-receptors in this setting. Accordingly, inhibition of CXCR4 and CXCR7 did not lead to any changes of gene (Figure 4E) and protein (Figure 4F) expression of the tight junction proteins in A_2B_–/– animals.

### Specific CXCR4- and CXCR7-Inhibition Dampens the Release of Inflammatory Cytokines by Controlling Intracellular Pathways

**Figure 5:**
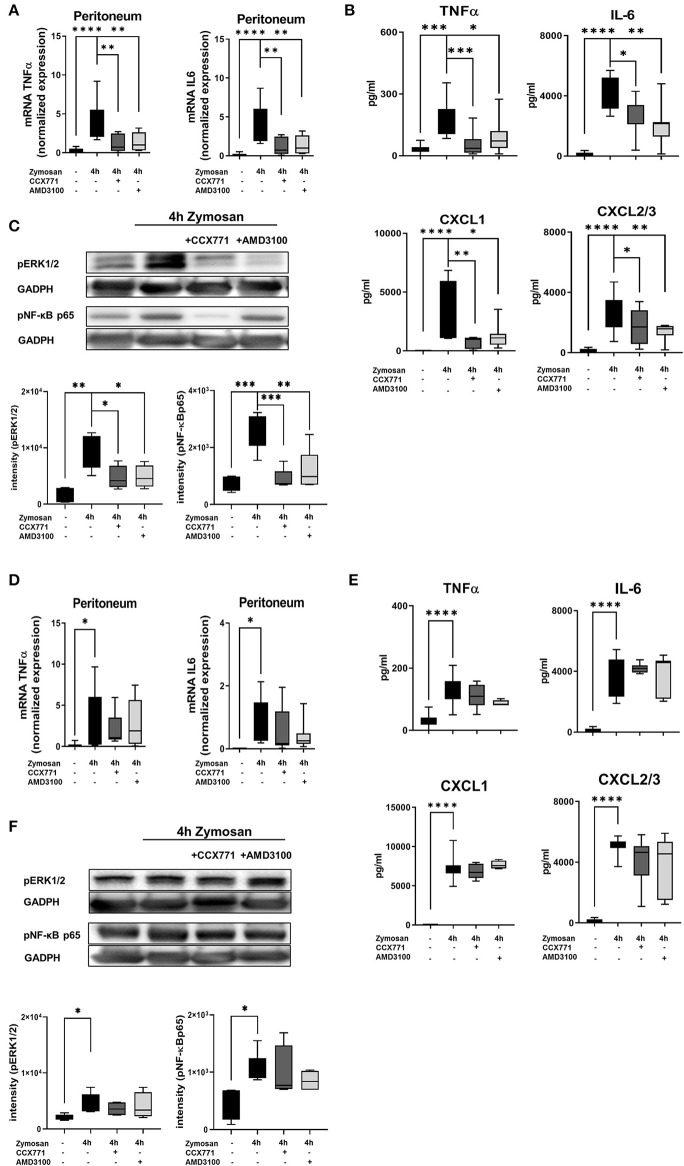
Impact of the SDF-1 receptors CXCR4 and CXCR7 on the release of inflammatory cytokines and intracellular pathways. **(A)** After the induction of peritonitis by zymosan, effects of AMD3100 or CCX771 on the gene expression of TNFα and IL6 were evaluated in peritoneal tissue of wild type and **(D)** A_2B_–/– mice. **(B)** The release of inflammatory cytokines (TNFα and IL6) and chemokines (CXCL1 and CXCL2/3) in the peritoneal cavity of wild type and **(E)** A_2B_–/– animals was detected. **(C)** Effects of CXCR4 and CXCR7 inhibition on the phosphorylation of intracellular ERK1/2 and NF-κB subunit 65 in peritoneal tissue of wild type and **(F)** A_2B_–/– animals 4 h after NaCl or zymosan with or without AMD3100- or CCX771 were determined. Blots are representatives of *n* = 3 experiments. Intensity of the blots was evaluated by ImageJ. For statistical analysis, one-way ANOVA was used with Bonferroni *post-hoc* test. Data are presented as box and whisker graph with error bars indicating the range from minimum to maximum value; *n* = 6–12; *p < 0.05; **p < 0.01; ***p < 0.001, and ****p < 0.0001.

To further define the protective role of CXCR4 and CXCR7 antagonism during acute inflammatory peritonitis, we evaluated the expression and release of inflammatory cytokines in peritoneal tissue and peritoneal lavage. Zymosan significantly increased gene expression of TNFα and IL6 in wild type animals, and both inflammatory cytokines were reduced after the administration of CCX771 and AMD3100 (Figure 5A). Based on these results, we evaluated the release of TNFα, IL6, and additionally CXCL1 and CXCL2/3 as the main PMN chemoattractants, in the peritoneal cavity. Zymosan increased all inflammatory cytokines and the inhibition of both receptors reduced the release significantly (Figure 5B), confirming and explaining our results from the PMN migration assay.

To identify the mechanism behind these findings, we determined the phosphorylation and therefore activation of the intracellular signaling proteins ERK1/2 and NF-κB p65. ERK1/2 activates the transcription factor NF-κB, which regulates the replication of cytokines (47, 48). Zymosan induced the activation of ERK1/2 and NF-κB p65, whereas AMD3100 reduced mainly the activation of ERK1/2 and CCX771 mostly the phosphorylation of the NF-κB subunit 65 (Figure 5C).

In A_2B_–/– animals, TNFα and IL6 gene expression increased after inflammation and the administration of AMD3100 and CCX771 did not lead to significant changes (Figure 5D). Correspondingly, AMD3100 and CCX771 failed to control the release of TNFα, IL6, CXCL1, and CXCL2/3 into the peritoneal cavity (Figure 5E) and did not influence the phosphorylation of ERK1/2 and NF-κB p65 in the peritoneal tissue of A_2B_–/– mice (Figure 5F).

### Functional Inhibition of CXCR4 and CXCR7 Dampens the Inflammatory Response During Polymicrobial Inflammation

**Figure 6:**
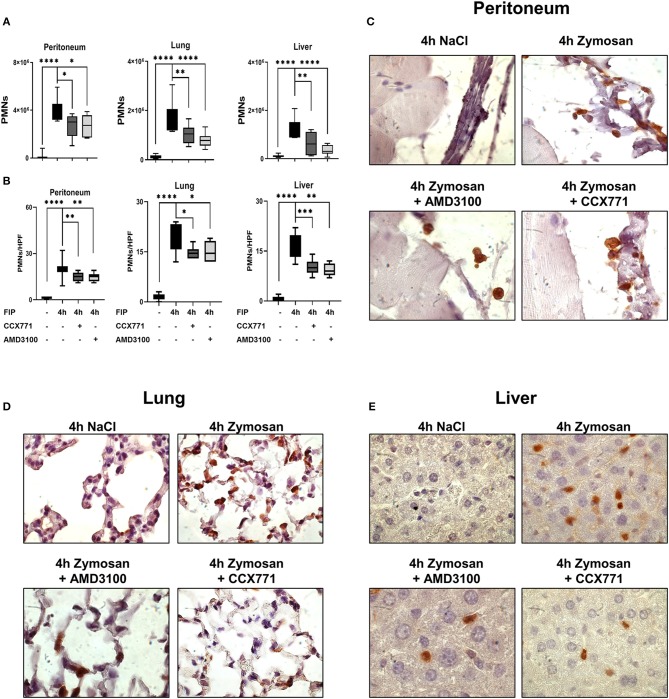
Pharmacological modulation of CXCR7 and CXCR4 dampens PMN accumulation during polymicrobial peritonitis. **(A)** In wild type animals, PMN counts in peritoneal, lung and liver tissue were evaluated by flow cytometry after injection of autologous feces and the effects of AMD3100 and CCX771 determined. Immonofluorescence staining marked PMNs brown and neutrophil infiltration was assessed and counted per high power field **(B)**, **(C)** in the peritoneum, **(D)** lung and **(E)** liver tissue at indicated conditions. Images are representatives of *n* = 3 experiments. For statistical analysis, one-way ANOVA was used with Bonferroni *post-hoc* test. Data are presented as box and whisker graph with error bars indicating the range from minimum to maximum value; *n* = 6–12; *p < 0.05; **p < 0.01; ***p < 0.001, and ****p < 0.0001.

To further verify the clinical impact of the inhibition of both SDF-1 receptors, we performed additional experiments with the injection of a fecal-solution to induce a polymicrobial inflammation. We determined PMN infiltration into the peritoneal lavage, lung and liver tissue by flow cytometry. In wild type mice, fecal solution led to increased PMN accumulation in the peritoneal cavity, lung and liver tissue (Figure 6A), whereas specific CXCR4 and CXCR7 antagonism significantly reduced PMN infiltration. Immunohistochemical blinded evaluation (Figure 6B) of the peritoneal tissue (Figure 6C), lung (Figure 6D), and liver (Figure 6E) confirmed our results.

**Figure 7:**
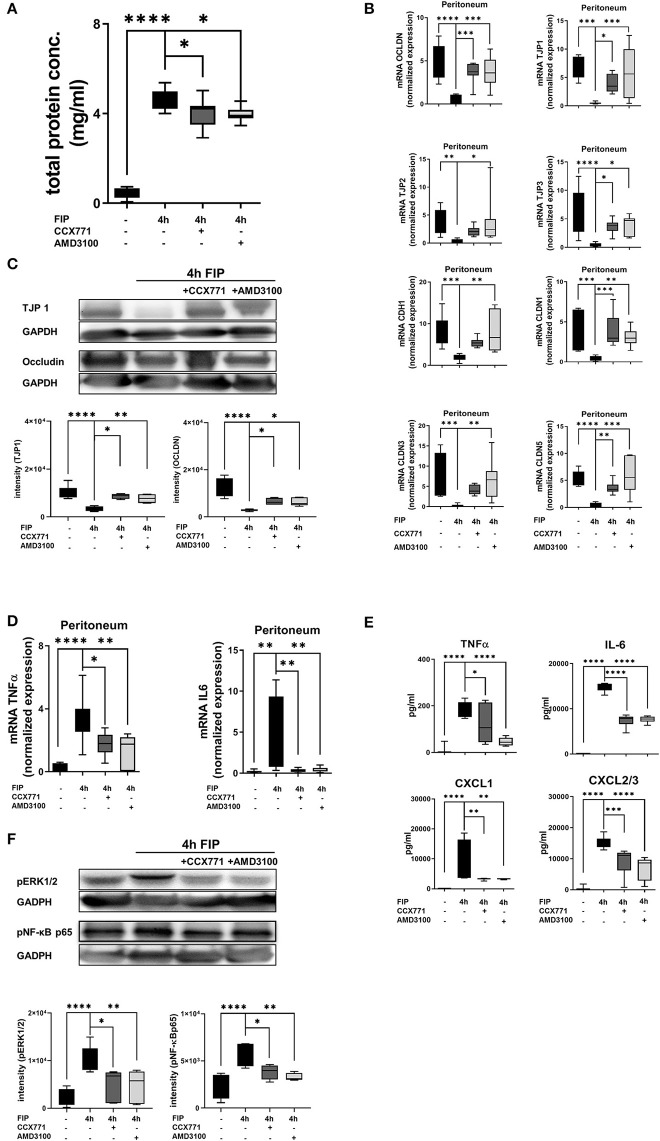
Impact of CXCR4 and CXCR7 inhibition on microvascular permeability and cytokine release during polymicrobial peritonitis. **(A)** Protein extravasation was evaluated in the peritoneal cavity 4 h after fecal solution in wild type animals (*n* = 8–12). **(B)** mRNA of tight junction proteins was measured in peritoneal tissue of wild type animals (*n* = 6–12). **(C)** Protein levels of TJP1 and occludin were quantified in peritoneal tissue (representative blot of *n* = 3 experiments) and also **(D)** gene expression of inflammatory cytokines TNFα and IL6 (*n* = 6–8). **(E)** TNFα, IL6, CXCL1, and CXCL2/3 release was determined in the peritoneal cavity at indicated time points and conditions (*n* = 8). **(F)** Effects of CXCR4- and CXCR7 blockade on the phosphorylation of ERK1/2 and NF-κB subunit 65 in peritoneal tissue of wild type were identified (representative blots of *n* = 3 experiments). Intensity of the blots was evaluated by ImageJ. For statistical analysis, one-way ANOVA was used with Bonferroni *post-hoc* test. Data are presented as box and whisker graph with error bars indicating the range from minimum to maximum value; *p < 0.05; **p < 0.01; ***p < 0.001, and ****p < 0.0001.

We also investigated the second hallmark of acute inflammation in the polymicrobial model, the microvascular leakage. Four hours after fecal injection into the peritoneal cavity of wild type animals, protein extravasation increased significantly. CCX771 and AMD3100 administration dampened protein extravasation and protected the cellular integrity (Figure 7A). To verify the impact of CXCR4 and CXCR7 on capillary barrier, we evaluated the expression of tight junction proteins (Figure 7B). After polymicrobial peritonitis, all tight junction proteins were significantly decreased. The inhibition of CXCR7 elevated gene expression of occludin, TJP1, TJP3, claudin 1, and 5. The selective CXCR4 antagonist ameliorated gene levels of occludin, TJP1, TJP2, TJP3, e-cadherin 1, claudin 1, 3, and 5 in fecal peritonitis. Protein analyses confirmed the results from gene expression with a pivotal effect of AMD3100 and CCX711 administration on the protein expression of tight junctions (Figure 7C).

Further on, we evaluated the expression and release of inflammatory cytokines in the peritoneal lavage. The administration of CCX771 and AMD3100 significantly reduced TNFα and IL6 gene expression in peritoneal tissue 4 h after the injection of autologous feces (Figure 7D). The detection of protein levels of TNFα and IL6 confirmed these findings. Additionally, the main PMN chemoattractants CXCL1 and CXCL2/3 were also significantly reduced after the inhibition of both receptors, supporting our results of PMN migration into the different organs (Figure 7E). To verify our findings, we evaluated the phosphorylation of ERK1/2 and NF-κB p65 in peritoneal tissue during fecal-induced peritonitis (Figure 7F), since ERK1/2 induces the activation of NF-κB p65, which controls the transcription of inflammatory cytokines (47, 48). Polymicrobial peritonitis activated ERK1/2 and NF-κB p65 signaling, whereas AMD3100 and CCX771 showed protective effects on the phosphorylation of both intracellular proteins.

### AMD3100 and CCX771 Enhances Barrier Integrity and Controls Cytokine Release *in vitro*

**Figure 8:**
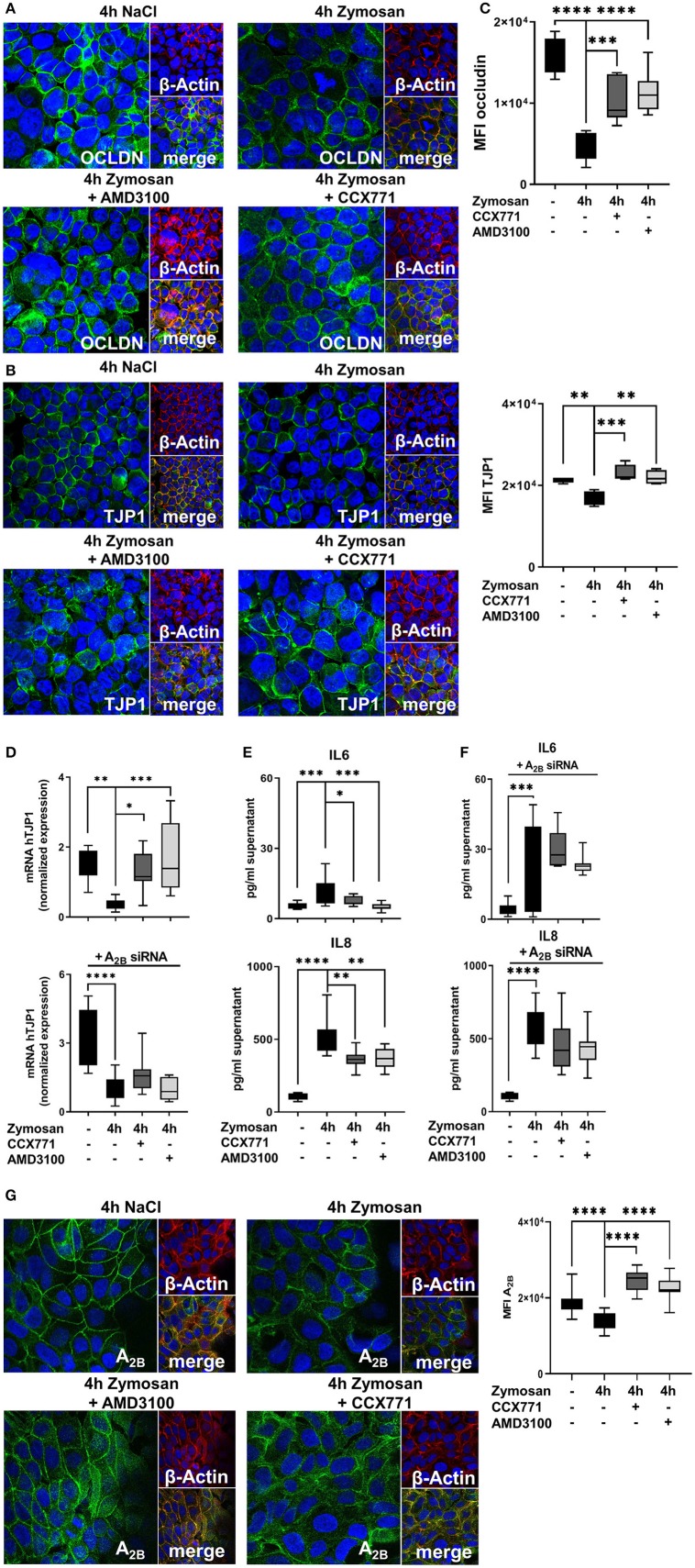
Effects of the adenosine receptor A_2B_ blockade on permeability and cytokine release *in vitro*. **(A)** Expression of occludin (OCLDN; green) and **(B)** tight junction protein 1 (TJP1; green) in human epithelial cells (β-Actin; red) 4 h after NaCl or zymosan with or without AMD3100 or CCX771 (63x original magnification). Images are representatives of *n* = 3 experiments. **(C)** Fluorescence intensity of occludin and TJP1 was measured at indicated conditions by using ImageJ (*n* = 8–12). **(D)** TJP1 expression and the effects of CXCR4 and CXCR7 inhibition after adenosine receptor A_2B_ knock down was evaluated (*n* = 8–10). **(E)** The inflammation-related release of human IL6 and IL8 from epithelial cells and **(F)** the effects of A_2B_-siRNA were determined in cell supernatants at indicated conditions (*n* = 8–12). **(G)** Protein levels of A_2B_ (green) in human H441 cells (β-Actin; red) at indicated conditions (63x original magnification). Image are representatives of *n* = 3 experiments. Fluorescence intensity was measured by ImageJ (*n* = 8–12). For statistical analysis, one-way ANOVA was used with Bonferroni *post-hoc* test. Data are presented as box and whisker graph with error bars indicating the range from minimum to maximum value; *p < 0.05; **p < 0.01; ***p < 0.001; ****p < 0.0001.

To support our data, we added *in vitro* experiments and determined the impact of CXCR4 and CXCR7 inhibition on human epithelial cells. To visualize the effects of a pharmacolocic inhibition of CXCR4 and CXCR7, we evaluated the protein expression of occludin and TJP1 by immunofluorescence. Confirming our *in vivo* data, immunofluorescence slides revealed a very high expression of occludin and TJP1 on epithelial cells. Zymosan installation reduced the expression of both proteins and specific pharmacologic inhibition of CXCR4 and CXCR7 improved the surface presentation of occludin (Figure 8A) and TJP1 (Figure 8B). Visual aspects were verified by the detection of the mean fluorescence intensities (MFI) of occludin and TJP1 (Figure 8C). Furthermore, zymosan induced a strong reduction of TJP1 gene expression, which significantly increased again after the inhibition of CXCR4 and CXCR7, confirming our *in vivo* data (Figure 8D). A_2B_-depletion by siRNA impeded the recovery of TJP1 expression after the treatment with both inhibitors. Additionally, we evaluated the release of IL6 and IL8 after zymosan stimulation. Zymosan increased the release of IL6 and IL8 by epithelial cells. These chemokine levels were significantly reduced after the administration of CCX771 and AMD3100 (Figure 8E). Depletion of A_2B_ abolished these protective effects on chemokine release (Figure 8F). These findings confirm our *in vivo* data and highlight the pivotal role of pharmacological inhibition of CXCR4 and CXCR7 in acute inflammation. The zymosan-induced reduction of the A_2B_ expression on H441 cells was significantly increased after the pharmacologic inhibition of CXCR4 and CXCR7 and confirmed our previous *in vivo* results (Figure 8G). To further investigate the impact of CXCR4 and CXCR7 inhibition on different cells, we performed additional experiments with the human intestinal epithelial cell line CaCo2. We evaluated the expression of occludin (Supplemental Figure 3A) and TJP1 (Supplemental Figure 3B) by immunofluorescence. Zymosan stimulation induced a significant reduction of both membrane proteins and AMD3100, respectively CCX771 significantly enhanced the expression of occludin and TJP1. The determination of the mean fluorescence intensities confirmed our *in vivo* and *in vitro* results (Supplemental Figure 3C). Additionally, gene levels of TJP1 were evaluated and the inhibition of both SDF-1 receptors induced a significant increase of TJP1 expression. Gene silencing of A_2B_ abolished the protective effects of CXCR4 and CXCR7 inhibition on TJP1 expression and confirmed our previous data with H441 cells and the A_2B_-dependent anti-inflammatory effects of blocking the SDF-1 receptors (Supplemental Figure 3D). The release of IL8 in the supernatant of CaCo2 cells after zymosan application was evaluated. Like our data with H441 cells, the CXCR4 and CXCR7 blockade reduced significantly the zymosan-induced IL8 liberation from the CaCo2 cells (Supplemental Figure 3E). The A_2B_-depletion by gene silencing abrogated the effects of AMD3100 and CCX771 on the release of IL8 and confirmed our previous *in vitro* data (Supplemental Figure 3F). To highlight the effects of CXCR4, respectively CXCR7 antagonism on the expression of A_2B_, we performed immunofluorescence experiments with CaCo2 cells. Zymosan stimulation decreased the surface expression of A_2B_ on CaCo2 cells, while the blockade of CXCR4 or CXCR7 augmented the A_2B_ expression (Supplemental Figure 3G).

## Discussion

The stromal cell-derived factor-1 and it's both receptors, CXCR4 and CXCR7, are expressed in various hematopoietic cells and non-hematopoietic tissue (25, 49–51). It is well-known, that SDF-1 and the CXCR4 receptor orchestrate the hematopoietic niche and regulate neutrophil release from the bone marrow into the circulatory system during inflammation (21). In the presented project, we determined the detailed role of CXCR4 and CXCR7 in acute septic inflammation concerning PMN migration and capillary leakage. Furthermore, our study provided new insights about the link between the SDF-1-CXCR4/CXCR7-axis and the adenosine receptor A_2B_ during acute peritoneal inflammation.

In the presented study, both receptors and the chemokine SDF-1 underwent an inflammation-related elevation of their expression in the peritoneum, liver and lung during zymosan- and polymicrobial-induced peritonitis. In line with our data, current studies demonstrated an increase of CXCR4 and CXCR7 after LPS stimulation (29, 50). Additionally, CXCR4 and CXCR7 were shown to play a detrimental role in inflammatory conditions like atherosclerosis (23), chronic hypoxia-related pulmonary hypertension (52), and ischemic cardiac disease (53, 54).

In a model of polymicrobial sepsis, blocking CXCR4 decreased sepsis-induced mortality (55). Additionally, Gosh et al. demonstrated that inhibition of CXCR4 reduced migration of cells by regulating cytoskeletal remodeling (56) and CXCR4 is considered as biomarker for peritoneal sepsis (57). Nevertheless, data on CXCR4 and sepsis is still conflicting. Delano and colleagues detected an increased mortality after inhibiting SDF-1 in a model of polymicrobial sepsis (58). In the presented study, inhibition of CXCR4 played a pivotal role in terms of PMN influx and microvascular permeability. To our knowledge, we are the first, who described a pivotal role of the inhibition of CXCR7 on PMN migration into the peritoneal lavage and various organs and, additionally, on capillary leakage during acute peritonitis and peritonitis-related sepsis. These findings are in line with the results of our previous publications, where blocking CXCR4 and CXCR7 influenced PMN migration and microvascular permeability in acute pulmonary inflammation (25, 29).

The string analysis, reflects protein-protein interactions in direct (physical) and indirect (functional) associations based on the actual literature. For this project, it showed a connection between SDF-1 and CXCR4/7 with the tight junction proteins over the mitogen-activated protein kinases (MAPKs). ERK1/2 belongs to the extracellular signal-regulated kinases, which are part of the MAPKs. ERK1/2 activates the transcription factor NF-κB. In the presented study, ERK1/2 and NF-κB p65 were activated in both peritonitis models. NF-κB controls the transcription of various genes, that are related with the release of inflammatory cytokines and barrier integrity (59–61). Selective antagonism of CXCR4 and CXCR7 dampened the phosphorylation and therefore activation of ERK1/2 and NF-κB p65, explaining the protective effects of CXCR4 and CXCR7 antagonism on tight junction proteins.

The expression of tight junction proteins is dampened by peritonitis, affecting barrier integrity and leading to tissue edema (62). In the presented study, we show for the first time, that antagonism of CXCR4 and CXCR7 restored microvascular permeability and increased the expression of essential tight junctions like tight junction protein 1 and occludin in peritoneal tissue. Furthermore, the treatment with AMD3100 and CCX771 enhanced the expression of e-cadherin, claudin 1, claudin 3, and claudin 5 in peritoneal tissue. These findings are in line with our previous publications from the impact of CXCR4 and CXCR7 inhibition on pulmonary permeability (25, 29), where blocking CXCR4 and CXCR7 stabilized and improved tight junctions like TJP1- and occludin. Further verifying our findings about the pivotal role of both SDF-1 receptors on stabilizing cellular integrity, inhibition of CXCR4 increased TJP-1, occludin, and claudin 5 in the blood/brain barrier in terms of an ischemic stroke and brain-specific metastasis in lung cancer (28, 61). Also, the inactivation of CXCL12 stabilized endothelial tight junction expression like TJP-1 and occludin in breast cancer metastasis (63).

Phosphorylation of ERK1/2 and NF-κB during inflammation initiates the transcription of inflammatory cytokines and chemokines, which induce the migration of neutrophils. CXCR4 and CXCR7 inhibition reduced gene transcription of cytokines in the peritoneum and the release of TNFα, IL6, CXCL1, and CXCL2/3 in the peritoneal lavage. In accordance with the presented data, CXCR4 and CXCR7 blockade decreased the release of inflammatory chemokines in acute pulmonary inflammation and human alcoholic hepatitis (25, 64).

In addition, signaling via adenosine and adenosine receptors decreased the cytokine release in inflammation as well (34, 65, 66). Adenosine receptors influence leukocyte migration and protect tissue from inflammatory damage (32, 67, 68). Numerous studies highlight the anti-inflammatory potential of the adenosine receptor A_2B_ in terms of acute inflammation or ischemia-reperfusion injury. These studies show the impact of the expression of the A_2B_ adenosine receptor in terms of myocardial infarction (69, 70), in acute pulmonary inflammation (71–73), and the expression of the receptor on the vascular endothelium (74, 75), and on different epithelial cell lines (32, 35, 76). Adenosine signaling stabilizes the cellular barrier and therefore microvascular permeability by inducing actin polymerization and changes in the cytoskeleton (77, 78). In the presented study, inhibiting CXCR4 and CXCR7 signaling enhanced the expression of the adenosine receptor A_2B_ and an increased phosphorylation of CREB. The A_2B_ adenosine receptor is known to activate CREB (46). CREB is a downstream signaling pathway of A_2B_ and a cellular transcription factor. The activation of CREB initiates mainly anti-inflammatory effects, for example stabilization of tight junction proteins (44) and inhibiting NF-κB (45). This increased phosphorylation of CREB through the adenosine receptor A_2B_ may explain the anti-inflammatory effects of the CXCR4, respectively CXCR7 inhibition on barrier integrity in the presented study. Lack of the adenosine receptor A_2B_ abrogated the protective effects of the pharmacological inhibition of CXCR4 and CXCR7. To our knowledge, we are the first detecting a link between the SDF-1/CXCR4/7 axis and functional A_2B_-receptor signaling in acute peritonitis. This finding is crucial, since patients on the intensive care unit may have altered adenosine receptor distribution and ligand affinity (37). Accordingly, the expression of the A_2B_-receptor should be investigated before the administration of the specific CXCR4 or CXCR7 antagonist.

## Conclusion

Our study identified a previously uncharacterized role of the SDF-1 receptors CXCR4 and CXCR7 in peritonitis and peritonitis-related sepsis. The inhibition of both receptors demonstrated anti-inflammatory effects on PMN-migration and tissue integrity and therefore revealed a pivotal anti-inflammatory role of pharmacological modulation of CXCR4 and CXCR7 in acute septic inflammation. The anti-inflammatory effects of the specific CXCR4 and CXCR7 inhibition depend on functional A_2B_-receptor signaling, enabling the identification of subgroups of patients, who would take advantage of this treatment.

## Data Availability Statement

The raw data supporting the conclusions of this article will be made available by the authors, without undue reservation, to any qualified researcher.

## Ethics Statement

All animal protocols were approved by the Animal Care and Use Committee of the University of Tübingen.

## Author Contributions

All authors made substantial contributions to this article. K-CN, CJ, RP, KS, CE, DK, and JG-T mainly contributed by participation in the data acquisition, analysis and interpretation. K-CN and FK contributed to the conception and design of the study, as well as the analysis and interpretation of the data. K-CN and FK wrote the manuscript.

### Conflict of Interest

The authors declare that the research was conducted in the absence of any commercial or financial relationships that could be construed as a potential conflict of interest.
