# Supplementary figures and images for: Inhibition of CXCR4 and CXCR7 Is Protective in Acute Peritoneal Inflammation
Source: Front Immunol. 2020 Mar 10;11:407. doi: 10.3389/fimmu.2020.00407 (PMC7076176; doi:10.3389/fimmu.2020.00407)

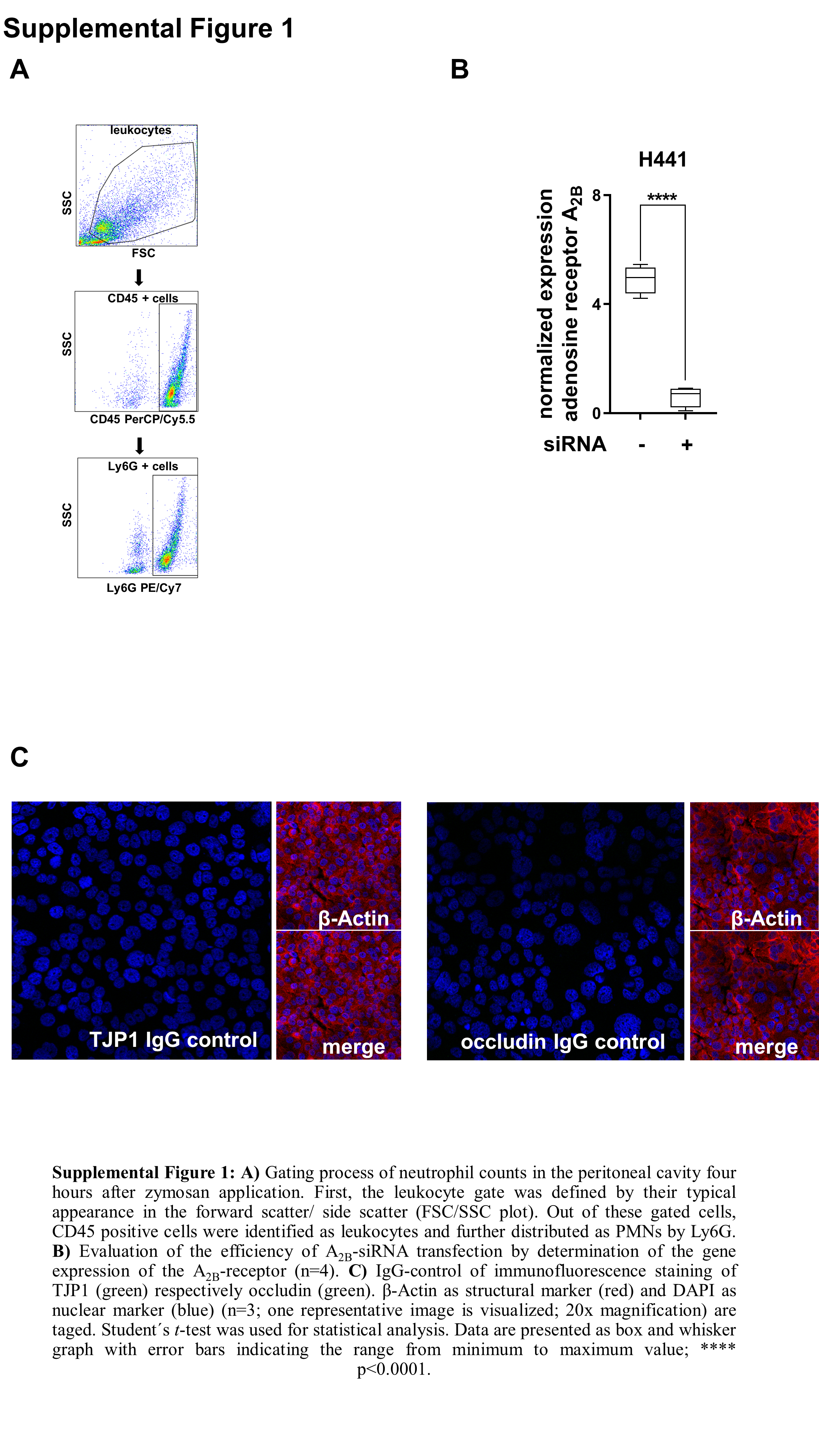

Supplement: Supplementary file 1 [file Image_1.TIF]

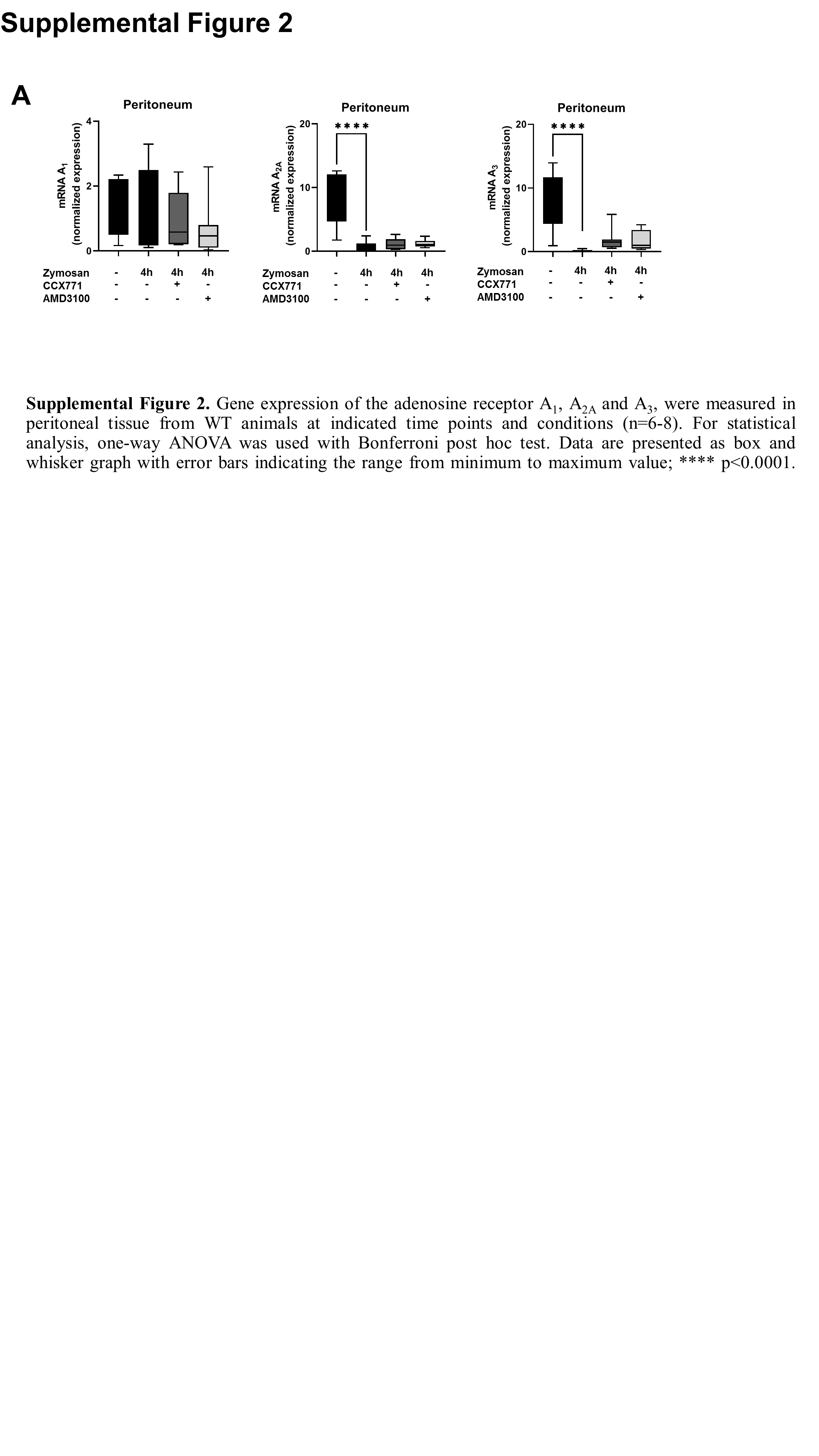

Supplement: Supplementary file 2 [file Image_2.TIF]
