# Supplementary material for: Inhibition of CXCR4 and CXCR7 Is Protective in Acute Peritoneal Inflammation
Source: Front Immunol. 2020 Mar 10;11:407. doi: 10.3389/fimmu.2020.00407 (PMC7076176; doi:10.3389/fimmu.2020.00407)
Supplement: Supplementary file 3 [file Image_3.pdf]

Supplemental Figure 3

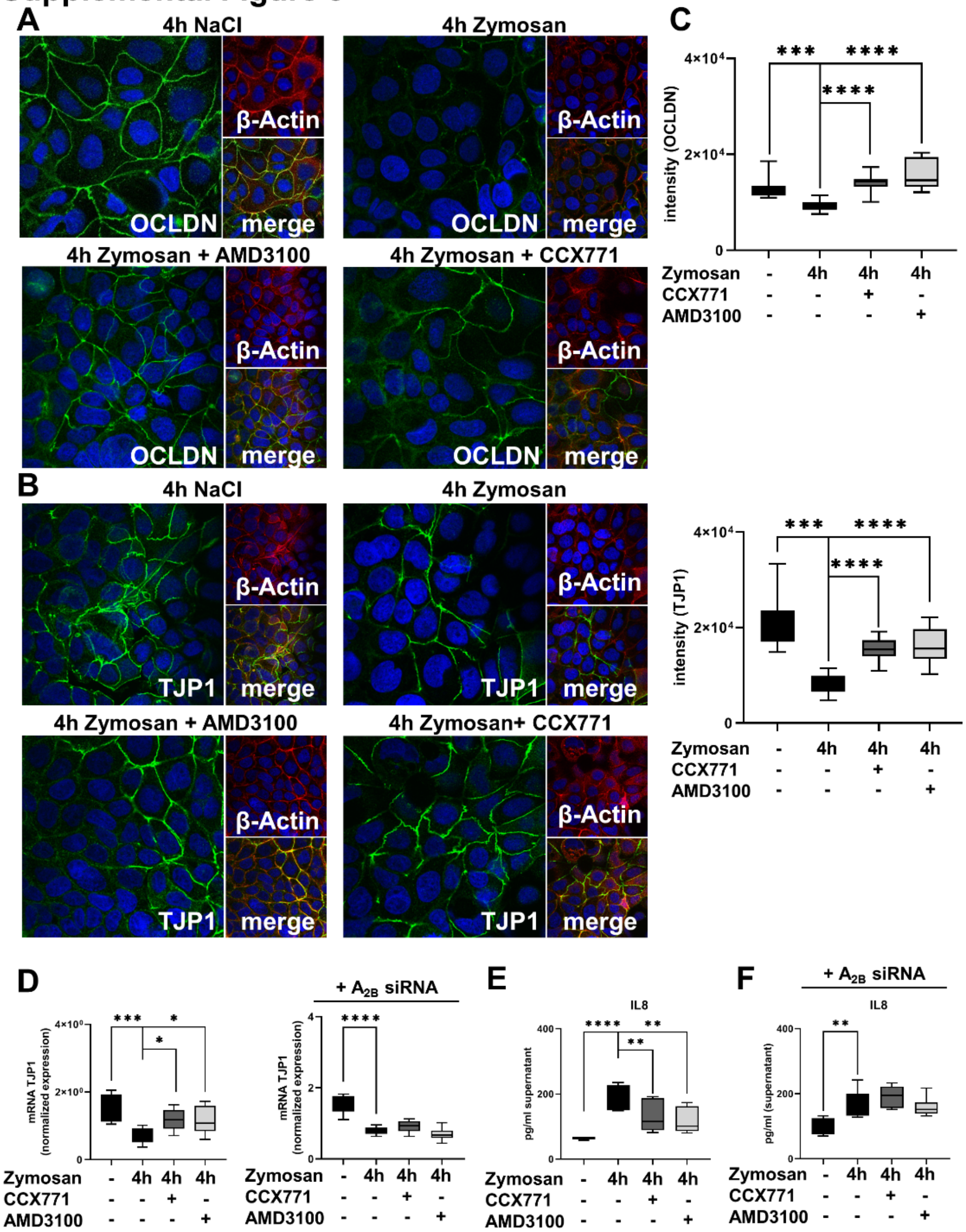

Supplemental Figure 3

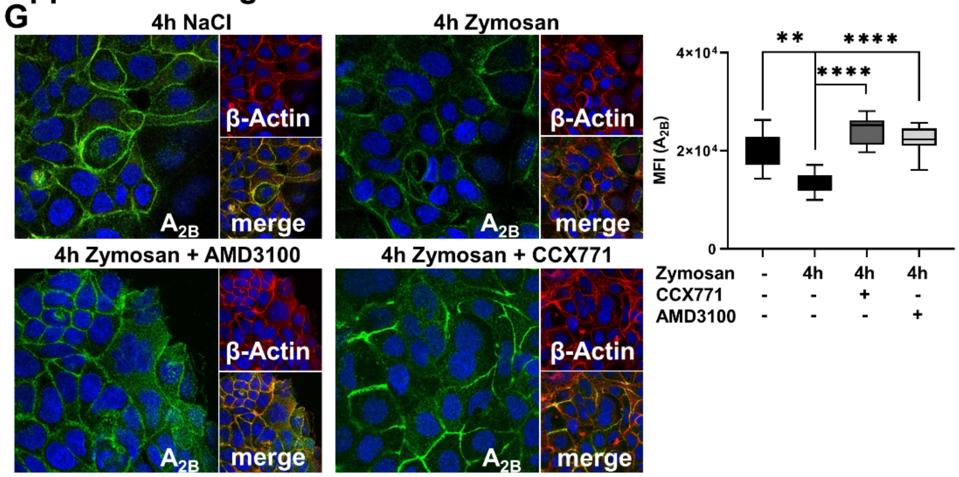

Supplemental Figure 3. Effects of the CXCR4 and CXCR7 blockade on epithelial integrity of human intestinal cell line CaCo2. A) Expression of occludin (OCLDN; green) and **B)** tight junction protein 1 (TJP1; green) in human epithelial CaCo2 cells (β-Actin; red) four hours after NaCl or zymosan with or without AMD3100 or CCX771 (63x original magnification). Images are representatives of n=3 experiments. **C)** Fluorescence intensity of occludin and TJP1 was measured at indicated conditions by using ImageJ (n=8-12). **D)** TJP1 expression and the effects of CXCR4 and CXCR7 inhibition without and after adenosine receptor A_{2B} knock down was evaluated (n=6-8). **E)** The inflammation-related release of human IL8 from CaCo2 cells (n=6-8) and **F)** the effects of A_{2B}-siRNA were determined in cell supernatants at indicated conditions (n=4-8). **G)** Presentation of the surface expression of A_{2B} (green) in human CaCo2 cells (β-Actin; red) at indicated conditions (63x original magnification). Image are representatives of n=3 experiments. Fluorescence intensity was measured by using ImageJ (n=8-12). For statistical analysis, one-way ANOVA was used with Bonferroni post hoc test. Data are presented as box and whisker graph with error bars indicating the range from minimum to maximum value; * p<0.05; ** p<0.01; *** p<0.001; **** p<0.0001.
